# Genomic characteristics and geographical distribution of uncultivated soil prokaryotes

**DOI:** 10.1186/s12864-026-13092-0

**Published:** 2026-08-04

**Authors:** Álvaro Rodríguez del Río, Yongxing Cui, India Mansour, Matthias C. Rillig

**Affiliations:** 1 Institute of Biology, Freie Universität Berlin, Berlin, 14195 Germany; 2 Berlin-Brandenburg Institute of Advanced Biodiversity Research (BBIB), Berlin, 14195 Germany

**Keywords:** Metagenomics, Uncultivated, Genome, Biogeography

## Abstract

Most soil prokaryotic species remain uncultivated, limiting our understanding of the terrestrial microbiome. Metagenomic sequencing, and particularly the study of metagenome-assembled genomes (MAGs), represents an unprecedented opportunity to characterize the genomic features and biogeography of uncultivated prokaryotic taxa at the large scale. Here, we analyze 40,039 genomic bins from cultivated and uncultivated soil taxa within the SMAG catalog, and examine the occurrence of uncultivated prokaryotes in 9,012 metagenomic samples from the Sandpiper resource. Compared to genera with cultivated representatives, uncultivated soil prokaryotes show smaller genomes, lower G + C content, tendency to acidophilic, non-alkaline, thermophilic and host-associated lifestyles, and slower growth rates, with the latter having the highest predictive power for cultivation status. Uncultivated soil microbes also show unique gene repertoires, characterized by a depletion of biosynthetic and motility genes. We also show that completely uncultivated genera are more abundant in tropical and arctic soils, indicating substantial hidden diversity in these regions. Our work emphasizes that current cultivation efforts systematically fail to capture a particular fraction of soil prokaryotic diversity, and provides guidelines for future cultivation strategies.

**Supplementary Information:**

The online version contains supplementary material available at 10.1186/s12864-026-13092-0.

## Introduction

Soil microbial diversity is the largest reservoir of biodiversity on Earth. Each gram of soil can harbor about 2 × 10^9^ microbial cells [1], and up to 3.3 × 10^9^ bacterial species may be present in soils across the world [2]. Yet, only a small fraction of this diversity has been cultivated [3–7]. Cultivation is important to allow characterization (e.g., physiology and metabolism), enables experimentation, and serves as a safeguard for microbial diversity [8]. However, recent metagenomic surveys suggest that over 85% of OTUs lack cultivated representatives, especially in environmental samples such as soil [9, 10], where fewer than 1% of soil-inhabiting species may have been cultivated so far [11], and that uncultivated taxa dominate soil communities worldwide [6].

Many more microbial species are observable using microscopy compared to culture-based methods, a phenomenon known as the Great Plate Anomaly [12]. It is frequently claimed that only 1% of environmental microorganisms are culturable; however, during the last decade this idea has been called into question [13, 14]. In fact, much more microbial diversity is likely potentially culturable, but not using easily accessible standard laboratory methods. Several factors may explain the difficulty in growing some soil-inhabiting species in isolation, including the challenge of reproducing essential features of their natural environments and widespread auxotrophies. Furthermore, typical microbiology workflows, including the use of standard agar growth media, incubation times that are too short to capture slow growers and settings that are unsuitable to resuscitate dormant cells also limit culturing efforts [5, 14–19].

Until these species are cultivated, they can only be studied with indirect methods. In particular, metagenome-assembled genomes (MAGs) are providing genomic sequences for thousands of novel species, redefining our view of microbial life [20] and soil diversity [21]. MAGs are also revealing important aspects of uncultivated microbes that can guide future cultivation efforts. For instance, doubling times, which can be predicted on MAGs with methods such as gRodon [22], indicate that cultivated species are biased towards rapid growers [22, 23]. MAGs have also revealed that uncultivated taxa generally have smaller genomes [24, 25]. The GenomeSPOT [26] method for estimating preferred growing conditions, suggests that uncultivated taxa are enriched in thermophilic, anaerobic, acidophilic and non-halophilic lineages. Additionally, symclatron [27], software for predicting lifestyles from genomic data, indicates that novel taxa are enriched in symbionts with reduced genomes and low G + C content. MAGs also revealed the functional gene content of uncultivated species, and have uncovered many relevant aspects of their metabolism, biosynthetic potential or defence systems [21, 28–30]. Recent efforts to describe the functional characteristics of uncultivated prokaryotes show that these taxa are enriched in pathways such as purine biosynthesis and NADH dehydrogenase, while being depleted in biosynthesis genes [31], a pattern also reported in MAGs from the human gut [24].

Environmental filtering strongly determines microbial community composition [32–34]; and soil prokaryotic populations show different traits than in other habitats, with larger genomes, higher G + C content, and different distribution of sigma factors and 16 rRNA gene copies [35]. Hence, soil uncultivated species may also present different characteristics than in other environments. Additionally, the genomic features of uncultivated taxa have not been addressed in combination, hindering the identification of the most important differences to cultivated species, and their geographical distribution has not been assessed. To obtain a general picture of the characteristics of uncultivated soil prokaryotes, we tested the genomic traits of 40,039 soil MAGs from the SMAG catalog [28], and reconstructed their geographical distribution through the taxonomic profiles of 9,012 soil samples from the Sandpiper database [11]. Overall, we observed similar attributes in uncultivated soil prokaryotes to those reported in previous analyses that were not specifically focused on soil taxa. However, we highlight the most important differences between cultivated and uncultivated soil species, and report novel aspects regarding their distinctive gene content and geographical distribution.

## Results

**Fig. 1:**
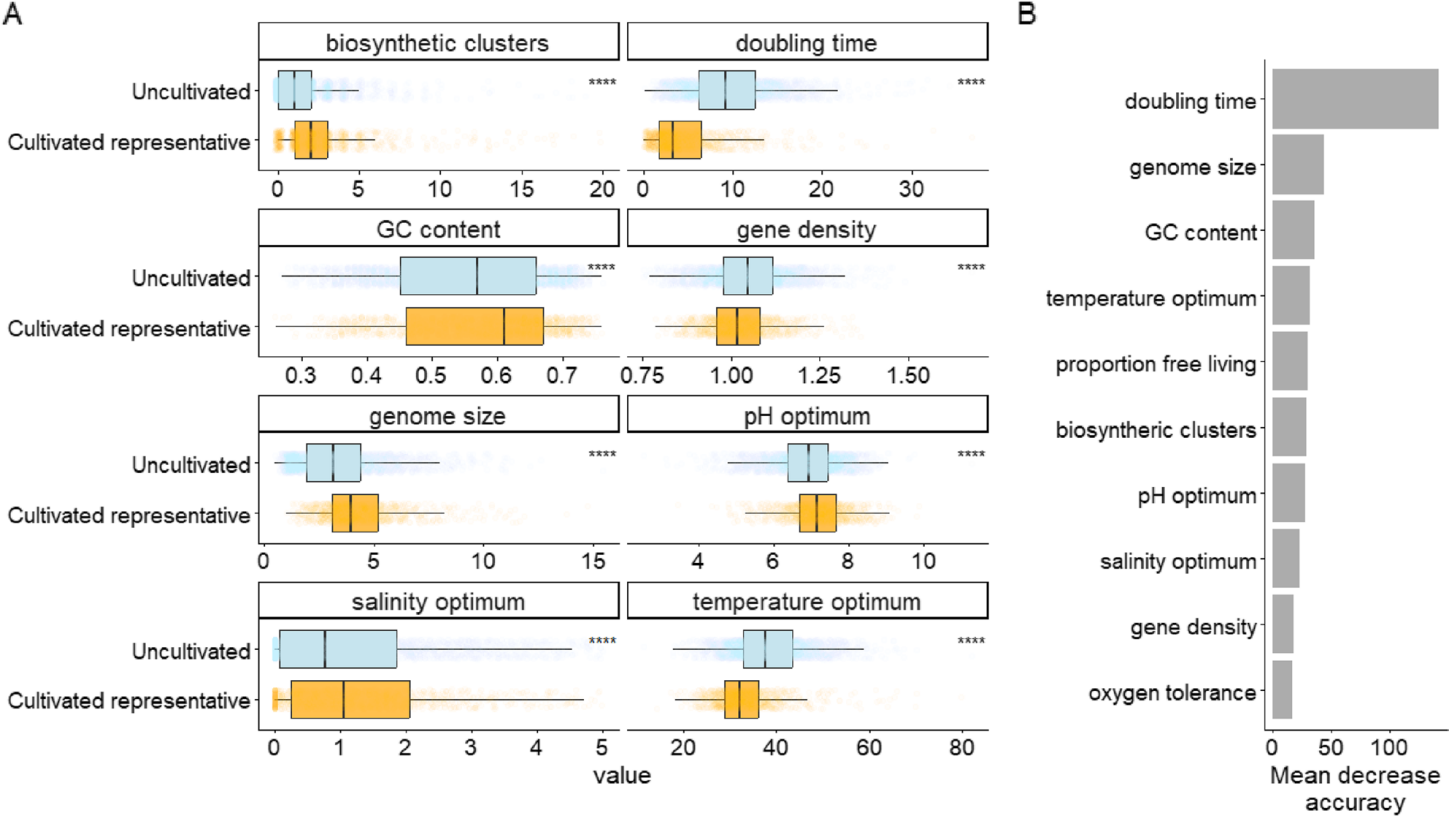
Genomic characteristics of cultivated and uncultivated soil prokaryotes. **A** Differences between predicted features for prokaryotic genera with cultivated representatives, and for completely uncultivated genera. Asterisks represent different significance levels obtained after a Two-sided Wilcoxon test comparing cultivated and non-cultivated genera; * indicate *p* ≤ 0.05, **p ≤ 0.01, ***p ≤ 0.001, and **** ≤ 0.0001. Biosynthetic clusters are represented in average numbers per genus. Doubling time is represented in hours. Gene density is represented as the average number of genes per Kbp. Genome size is represented in Mbps. Temperature is represented as Celsius degrees. Salinity is represented in w/v NaCl. **B** Mean decrease in accuracy in a random forest model for each of the variables tested

On average, genomes from soil uncultivated genera (which we defined as those not having representative genomes in RefSeq, which mostly contain genomes from isolates) are smaller than those from genera with cultivated representatives, with almost 1 Mbp difference (3.4 Mbp vs. 4.3, Wilcoxon test *p* = 3.9e-52). Uncultivated soil taxa also present higher gene density (Wilcoxon test *p* = 1.7e-13), lower G + C content (Wilcoxon test *p* = 1.4 e-5), and have longer predicted doubling times: soil species with cultivated representatives divide every 4.7 h on average, whereas non-cultivated species divide every 10 h (Wilcoxon test *p* = 6.4 e-199). We also confirmed previous predictions on the lifestyles of uncultivated species. Soil uncultivated taxa are enriched in symbionts, with 75% predicted to be free-living, compared with 95% of species with cultivated representatives (Chi square *p* = 6.11 e-46). They also harbor fewer biosynthetic gene clusters (Wilcoxon test *p* = 1.2 e-65), indicating a more restricted capability for secondary-metabolite biosynthesis (Fig. 1A, Fig. S1).

Cultivated and uncultivated species are also predicted to differ in environmental preferences, as previously reported. We confirmed that uncultivated soil taxa tend to prefer lower salinity (1.27 vs. 1.64 w/v NaCl, Wilcoxon test *p* = 1.7 e-9) and more acidic conditions (pH = 6.86 vs. 7.13, Wilcoxon test *p* = 7.2e-16). We also observed different temperature preferences, with novel taxa having an optimal growth temperature 7 °C higher (38.4 °C vs. 33.2 °C, Wilcoxon test *p* = 7.8e-93. Figure 1A, Fig. S1). Similarly, as in previous analyses, we observed oxygen tolerance differences: only 65% of genomes from uncultivated genera are predicted to be oxygen-tolerant, compared with 84% among genera with cultivated representatives (Chi square *p* = 4.4 e-32).

Given these differences, cultivation status can be accurately predicted by random forest models (AUC = 0.89). The most important predictor is doubling time (Fig. 1B), suggesting that enrichment for slow-growing bacteria may be especially important for culturing soil prokaryotes. Doubling time correlates with other features: slow-dividing taxa tend to have higher optimum growing temperatures (Spearman’s *r* = 0.42, *p* = 2e-158), lower oxygen tolerance (Wilcoxon test *p =* 2.4 e-77), lower G + C content (Spearman’s *r* = -0.22, *p* = 7e-41), and a higher probability of being non-free living (Wilcoxon test *p =* 1.6 e-19), as generally predicted for uncultivated lineages (Figs. S2 - S4). For evaluating the effect of our cultivated status definition, we repeated these analyses using a less restrictive threshold (i.e. < less than 10% representatives from non-reference genomes), obtaining similar results (Fig. S5).

**Fig. 2:**
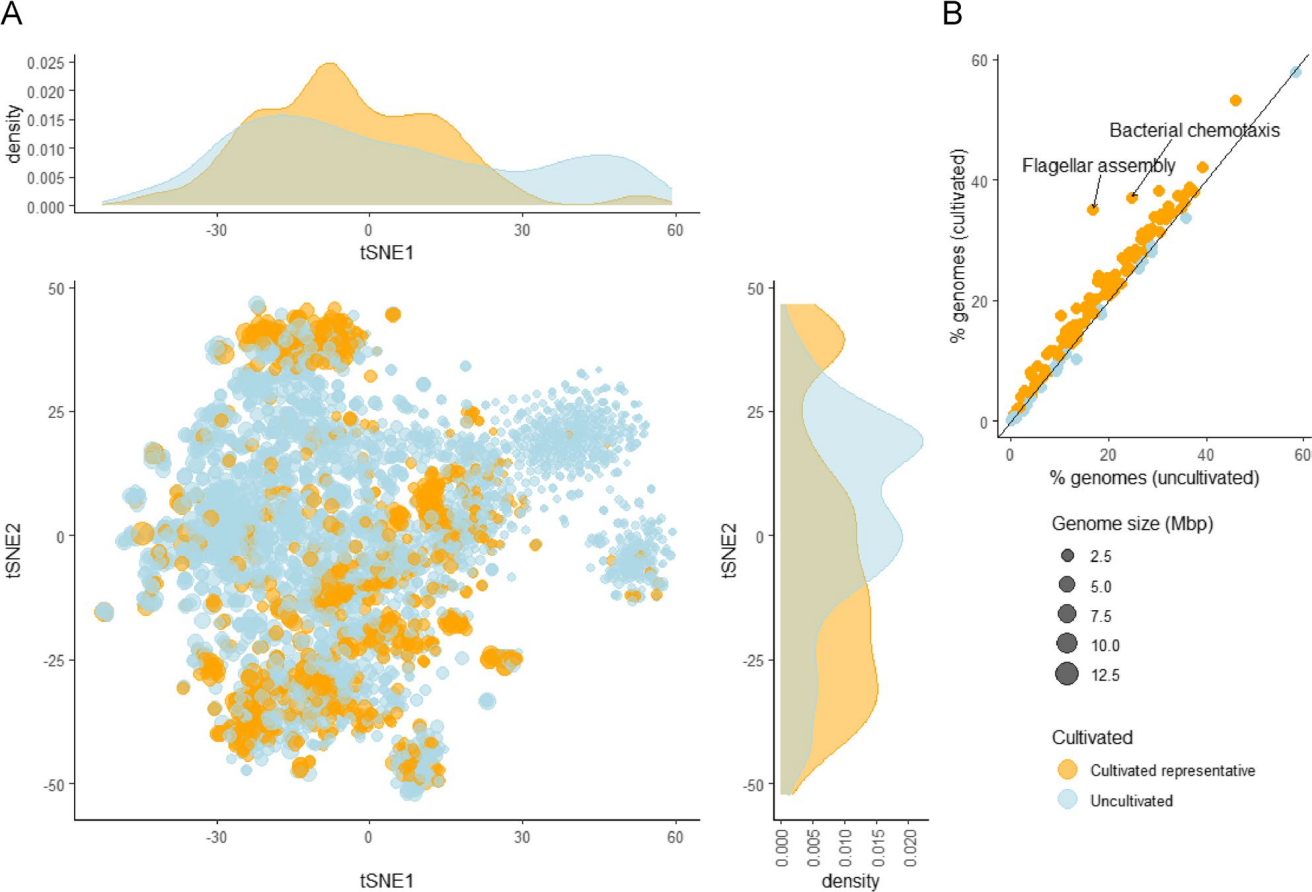
Functional characteristics of uncultivated and cultivated soil prokaryotes. **A** tSNE analysis on the distribution of KEGG pathways across soil prokaryotic genera. On the sides, density plots indicating the distribution of cultivated and non-cultivated genera. **B** Average KEGG pathway conservation per genus. The black line indicates the diagonal. We highlight routes with important differences in conservation between cultivated and non-cultivated taxa. Routes with higher conservation in cultivated genera are colored in orange, routes with higher conservation in uncultivated genera in blue

 Soil uncultivated taxa dominate the functional space in a t-distributed stochastic neighbor embedding (*t*-SNE) analysis (Fig. 2A) which mirrors the prokaryotic phylogeny (Fig. S6). For example, Patescibacteria (as also reported by [36]) and Nanoareota, both mostly uncultivated host-associated taxa, form clear functional clusters characterized by lower frequencies of central metabolism genes, such as amino acid metabolism (Wilcoxon test *p* = 0) or carbohydrate metabolism (Wilcoxon test *p* = 2.5e-240). Similarly, Thermoproteota genomes are characterized by lower frequencies of antibiotic resistance (Wilcoxon test *p* = 1e-125) and biofilm-formation genes (Wilcoxon test *p* = 7e-64) compared with other taxa.

Taxa with no cultivated representatives are systematically depleted in genes within functional pathways. For instance, 16 folate biosynthesis, 15 arginine biosynthesis and 10 lysine biosynthesis genes are depleted in genera with no cultivated representatives (FDR < 0.05). The most striking differences in the gene content of soil cultivated and uncultivated taxa remain in cell motility and chemotaxis: among 52 flagellar assembly genes and 26 chemotaxis genes, 42 and 15, respectively, were enriched in taxa with cultivated representatives (FDR < 0.05; Fig. 2B).

**Fig. 3:**
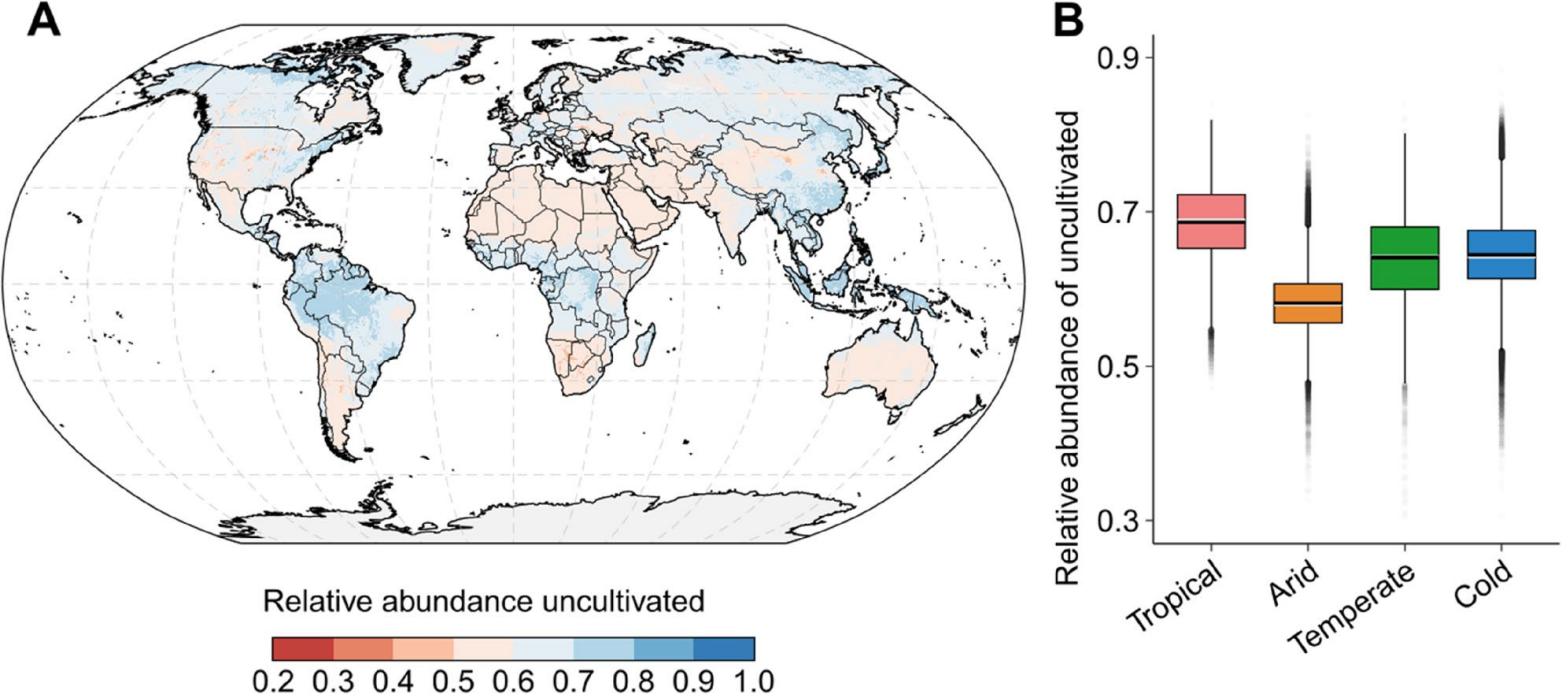
Geographical distribution of uncultivated taxa. **A** Predicted distribution of the relative abundance of uncultivated prokaryotic species. **B** Relative abundance of uncultivated genera in tropical, arid, temperate and cold soils. The climatic zones are delineated using the global Köppen-Geiger classification of climate [37]

We also predicted their global distribution at a 0.1° × 0.1° resolution using Random Forest models and 24 environmental predictors (Supplementary Table 1), which revealed that uncultivated taxa dominate microbial soil communities across the world (Fig. 3A), and are especially abundant in tropical and Arctic regions (Fig. 3B). The relative abundance of uncultivated taxa was best explained by CaCO3 concentration (Fig. S7), characteristic of dry, alkaline and low nutrient soils, which correlates negatively with the relative abundance of uncultivated prokaryotes (Spearman *r* = -0.08, *p* = 1.3 e-7).

## Discussion

In this study, we apply an integrative approach to summarise the characteristics and distribution of uncultivated taxa in the most biodiverse habitat on Earth. This work represents a comprehensive comparison of the traits of cultivated and uncultivated prokaryotes, and the first focused on soil taxa. Additionally, we describe the geographical distribution of uncultivated taxa worldwide.

Consistent with previous surveys not focused specifically on soil, we highlight that uncultivated soil genera systematically have AT-rich and small genomes, are more commonly host-associated, grow more slowly, and prefer higher temperatures, more acidic, less alkaline and more anoxic conditions than cultivated species [22, 24, 26, 27, 38]. Overall, taxa that have not been cultured present very particular features, indicating that there is a need not only for increasing cultivation efforts, which may have been lagging in soil compared to other environments, but also for adopting alternative cultivation strategies.

The most important difference between cultivated and uncultivated soil prokaryotes is their doubling time, as already suggested for MAGs from other habitats [22], with uncultivated prokaryotes taking about twice as long to divide as taxa with cultivated representatives. Longer doubling times were strongly associated with higher optimal growth temperature, in line with observations from marine environments [39], and with other traits such as anoxygenicity, which are all predicted features of uncultivated taxa. These longer doubling times suggest that penalizing fast-growing species in cultures may be one of the most effective strategies for increasing cultivation rates of soil prokaryotes. That may be achieved, for instance, by extending incubation times [15] or by using alternatives to nutrient-rich media, which favour faster-growing taxa [15] and penalize oligotrophs, which generally grow more slowly [40].

Another particularly important difference between taxa with cultivated representatives and novel taxa is the preferred temperature (as also noted by [26]), highlighting the need to find better methods for culturing thermophiles. Uncultivated soil taxa are predicted to grow at higher temperatures, and may become increasingly relevant under global warming [41], highlighting the importance of their characterization. Soil taxa adapted to high temperatures also have smaller genomes (Figs. S2, S3), consistent with previous observations in thermophilic bacteria [42]. Hence, methods for culturing thermophilic taxa, which have already shown success [43–45] are likely to be particularly relevant for many uncultivated soil prokaryotes. Uncultivated taxa are also anoxic more frequently, which reflects the difficulty of attaining anoxic conditions in cultivation, and highlights the need for conducting additional attempts [46, 47].

We also got similar predictions as [26] regarding the preferred salinity and pH conditions of uncultivated taxa. Uncultivated soil taxa prefer less alkaline soils than taxa with cultivated representatives. Previous work has shown that salt treatments can increase the relative abundance of conditionally rare novel taxa [48], suggesting that novel taxa adapted to high salinities may be common but generally low in abundance. We also predict that acidophiles are prevalent among uncultivated taxa, indicating that targeting these species, which has also been attained in some cases [49], may be a relevant approach.

Uncultivated soil species also have smaller genomes than those with cultivated representatives, as previously reported [24, 25, 27], and this reduction is coupled with more limited metabolic capabilities. We also predict that uncultivated soil taxa are enriched in host-associated lineages, which usually have smaller genomes, in agreement with patterns observed across the prokaryotic phylogeny [27]. Hence, co-culturing approaches seem to be relevant for culturing a substantial fraction of uncultivated soil prokaryotic species [17–19]. Moreover, uncultivated taxa have high gene densities, which is typical of auxotrophs and intracellular organisms [16]. However, host-association does not fully explain the streamlined genomes of uncultivated taxa; among free-living taxa, uncultivated genera have smaller genomes (Wilcoxon test *p* = 8.7 e-7).

We additionally observe lower G + C content in the genomes of uncultivated soil taxa, as also reported in the human gut [24]. Interestingly, G + C content correlates with growth temperature, an association that has been debated [50] but was recently confirmed at large scale [51]. G + C content is also lower in host-dependent taxa, as previously noted [52].

Uncultivated soil taxa also have a distinctive functional repertoire characterized by a more limited metabolic potential. Oligotrophy has already been described to be prevalent in soils [40] and has been observed in several uncultivated groups [24, 31]. Uncultivated soil taxa seem to be especially depleted in cell motility and chemotaxis genes, suggesting a general tendency towards immotility, which is also typical of oligotrophs [53], or stronger associations with hosts [52]. Chemotaxis is also important in biofilm-forming bacteria, which are known to be underrepresented in culture collections [15].

Our analysis of the biogeography of uncultivated taxa across the globe further reveals their environmental distribution and identifies hotspots for culturing efforts. Uncultivated prokaryotes dominate soil communities globally, as also highlighted in [6], even despite the fact that current reference databases still miss many uncultivated microbes [10]. Tropical and Arctic soils show higher abundances of uncultivated taxa, even though Arctic regions are not typically regarded as biodiversity hotspots [54], highlighting these habitats as promising targets for large-scale cultivation initiatives.

Overall, we provide the first integrative analysis on the genomes of cultivated and uncultivated soil prokaryotes, highlighting doubling times as the most important difference between both groups. We also show that uncultivated soil taxa are enriched in non-motile species and are particularly abundant in tropical and Arctic habitats, highlighting these regions as prime targets for future culturing efforts.

## Methods

### Genomic catalog and uncultivated definition

We ran our predictions on a collection of 40,039 soil MAGs from the SMAG catalog, which gathers 3,662 genera from 104 prokaryotic phyla [28]. We considered as uncultivated those genera with no reference genome in GTDB r214 [55], therefore highlighting whole lineages that may be inherently difficult to cultivate. Even after this restrictive definition, 2,581 genera (70.4%) spanning 85 phyla, are uncultivated (Fig. S8), with 52 phyla only consisting of uncultivated genera. Overall, Patescibacteria was the phylum presenting more uncultivated genera (410).

### Genomic feature predictions

We leveraged the SMAG catalog to make genome-based predictions, which we averaged per genus before running the comparisons to correct for taxon sampling biases, and to highlight the characteristics of whole genera that may be inherently difficult to cultivate, despite species level variations. Genome size was estimated from MAG size, correcting for completeness by dividing by the average completeness of each genus. G + C content was also estimated from MAGs with a custom script. Growing conditions were estimated with GenomeSPOT [26]. Lifestyle predictions were conducted using symclatron [27]. For running gene predictions, we ran Prodigal [56] (-p meta and -f gff parameters) on the contig predictions. Eggnog-mapper v2 [57] was run for obtaining the functional annotation of the genes (--itype proteins --block_size 0.4 options). For calculating doubling time we used the gRodon software [22], considering genes with KOs annotated as “subunit ribosomal” as ribosomal genes. Biosynthetic gene pathway number was predicted with antiSMASH [58] in the original SMAG publication. After averaging per genus, genera with more than 50% genomes predicted to be oxygen tolerant and free living were labelled as so. Random forest predictions were performed with the `randomForest` R library.

### Functional analysis

For calculating the distribution of gene functions across lineages, we computed, for each gene annotation *g* (i.e. KEGG Orthologs (KO) and KEGG pathways) and genus (*l*), a conservation value (*C*), which represents the proportion of genomes from the genus coding for the gene annotation, corrected by MAG completeness:

1 $$C \left(g, l\right)=\frac{\sum{Ii}}{\sum{ci}}$$

We used KEGG pathway *C* values for all genera to compute the tSNE analysis using the sklearn.manifold python library. We only included genera with at least 10 genomes available. Gene differential abundances were performed using Wilcoxon tests on KO *C* values, and correcting for multiple testing with the FDR method, using the statsmodels and scipy python libraries.

### Geographical mapping

Prokaryotic detections were retrieved from the Sandpiper database [11]. Samples coming from soil metagenomes were identified in the sample metadata from the NCBI.

#### Environmental variables

To predict the global distribution of relative abundance of uncultivated prokaryotic species and identify the potential drivers, we retrieved a variety of climatic, topographic, vegetation, and soil variables from multiple data products at a relatively fine spatial resolution (see Table S1 for more details). We obtained a total of 24 environmental variables with coordinates matching the site locations of our gene dataset. Furthermore, we resampled and reprojected all covariate map layers to a unified pixel grid in EPSG:4326 (WGS84) at a resolution of 0.1 × 0.1° (approximately 111 km2 at the equator) for predicting global distributions of relative abundance of uncultivated taxa. Layers with a higher original pixel resolution were down-sampled using a mean aggregation method, while none of the original layers had pixels below a resolution of 0.1 × 0.1°. 

#### Upscaling predictions

We trained the Random Forest (RF) models by including all 24 environmental variables using the “RandomForest” R package. RF is an ensemble learning technique developed by Breiman [59] to improve the classification and regression-tree method by combining a large set of decision trees. A 10-fold cross-validation was conducted to determine the final models and the potential importance of the variables using the “rfcv” function. The level of model significance and cross-validated R2 were assessed with 1500 permutations of the response variable using the “A3” R package. The constructed RF regression models for predicting relative abundance of uncultivated taxa explained 64.3% of the variations in relative abundance (Fig. 3). Furthermore, we identified the relative importance of climate, topography, vegetation, and soil properties in explaining the variations in relative abundance of uncultivated taxa by obtaining the values of increased mean squared error (Increase in MSE, %) for each potential predictor in our constructed RF models using the “rfPermut” R package (Fig. 3). Similarly, we assessed the statistical significance of each predictor on the response variables using the “rfPermute” R package. 

#### Global mapping

We ultimately mapped the global distribution of relative abundance of uncultivated taxa using the RF regression models at a resolution of 0.1 × 0.1° (Fig. 3). Meanwhile, we obtained additional global mapping of prediction uncertainty (standard deviation of predictions) in relative abundance of uncultivated taxa at a resolution of 0.1 × 0.1° using the “apply” function (Fig. S9). Areas of water, permanent wetlands, and snow/ice based on the MODIS land-cover map (https://modis-land.gsfc.nasa.gov/landcover.html*)* were excluded to avoid non-soil land influencing our predictions for global land areas. We then summarized the relative abundance of uncultivated taxa across climate classification (Fig. 3B) by overlapping our predictions with the global maps of the Köppen-Geiger classification of climate [37].

## Supplementary Information

Supplementary Material 1: Fig S1. Differences between predicted features for prokaryotic genera with cultivated representatives, and for completely uncultivated genera (violin plots). Figure S2. Spearman correlations across predicted features in the SMAG catalog. Non-significant correlations are not shown. Fig S3. Correlations across predicted continuous genomic features in the SMAG genomic catalog. Fig S4. Differences in genomic characteristics between free living and host associated genera, and between oxygen tolerant and non-tolerant genera. Fig S5. Genomic characteristics of cultivated and uncultivated soil prokaryotes, taking as uncultivated those genera with less than 10% reference genomes. Fig S6. tSNE analysis on the distribution of KEGG pathways across soil prokaryotic genera, colored by phylum. Fig S7. Key environmental factors driving global patterns of estimated uncultivated taxa. Fig S8. Distribution of the proportion of reference genomes per genus in the GTDB r214 database. Fig S9. Global distribution of the uncertainty in prediction of uncultivated taxa.

Supplementary Material 2. Table S1. Description of data for the 24 predictors of soil uncultivated proportion on a global scale.

## Data Availability

Taxonomic profiles were downloaded from the Sandpiper database (https://sandpiper.qut.edu.au/). Soil MAGs were retrieved from the SMAG catalog, downloaded from (10.5281/zenodo.7341719). The code and data for running the statistical tests and figures can be accessed at https://github.com/AlvaroRodriguezDelRio/uncultivated_soil_taxa_genomic_chars.
