# Supplementary material for: Genomic characteristics and geographical distribution of uncultivated soil prokaryotes
Source: BMC Genomics. 2026 Aug 4;27:655. doi: 10.1186/s12864-026-13092-0 (PMC13445660; doi:10.1186/s12864-026-13092-0)
Supplement: Supplementary file 1 [file 12864_2026_13092_MOESM1_ESM.docx]

**Supplementary figures**

*Figure S1.* *Differences between predicted features for prokaryotic genera with cultivated representatives, and for completely uncultivated genera (violin plots). Asterisks represent different significance levels obtained after a Two-sided Wilcoxon test comparing cultivated and non-cultivated genera; * indicate p* ≤ 0.05*, **p* ≤ 0.01*, ***p ≤* 0.001, and ***** ≤* 0.0001*. Biosynthetic clusters are represented in average number per genus. Doubling time is represented in hours. Gene density is represented as the average number of genes per Kbp. Genome size is represented in Mbps. Temperature is represented as Celsius degrees. Salinity is represented in w/v NaCl.*

*Figure S2. Spearman correlations across predicted features in the SMAG catalog. Non-significant correlations are not shown.*

*Figure S3. Correlations across predicted continuous genomic features in the SMAG genomic catalog.*

*Figure S4. Differences in genomic characteristics between free living and host associated genera, and between oxygen tolerant and non-tolerant genera. Asterisks represent different significance levels obtained after a Two-sided Wilcoxon test comparing cultivated and non-cultivated genera; * indicate p* ≤ 0.05*, **p* ≤ 0.01*, ***p ≤* 0.001, and ***** ≤* 0.0001*. Biosynthetic clusters are represented in average number per genus. Doubling time is represented in hours. Gene density is represented as the average number of genes per Kbp. Genome size is represented in Mbps. Temperature is represented as Celsius degrees. Salinity is represented in w/v NaCl*

*Figure S5.* *Genomic characteristics of cultivated and uncultivated soil prokaryotes, taking as uncultivated those genera with less than 10% reference genomes. A) Differences between predicted features for prokaryotic genera with cultivated representatives, and for completely uncultivated genera. Asterisks represent different significance levels obtained after a Two-sided Wilcoxon test comparing cultivated and non-cultivated genera; * indicate p* ≤ 0.05*, **p* ≤ 0.01*, ***p ≤* 0.001, and ***** ≤* 0.0001*. Biosynthetic clusters are represented in average number per genus. Doubling time is represented in hours. Gene density is represented as the average number of genes per Kbp. Genome size is represented in Mbps. Temperature is represented as Celsius degrees. Salinity is represented in w/v NaCl. B) Mean decrease in accuracy in a random forest model for each of the variables tested.*

*Figure S6. tSNE analysis on the distribution of KEGG pathways across soil prokaryotic genera, colored by phylum. Phyla with less than 50 representative genera were labelled as “Other”.*

*Figure S7. Key environmental factors driving global patterns of estimated uncultivated taxa. Random-forest models identify the relative contributions of each environmental predictor in explaining the variations of uncultivated taxa (64.3% explained (cross-validated R^2^)). A total of 24 environmental factors were used as potential predictors of uncultivated taxa. Climatic factors: mean annual temperature (MAT), mean annual precipitation (MAP), temperature seasonality (TSEA), precipitation seasonality (PSEA), and aridity index (AI). Topographical factors: elevation and slope. Vegetation factors: Shannon diversity enhanced vegetation index (Shannon EVI), leaf area index (LAI), gross primary production (GPP), aboveground biomass (AGB), litter production (Litter), and belowground biomass (BGB). Soil factors: Depth to Bedrock, soil moisture content (Moisture), cation exchange capacity (CEC), soil pH (pH), soil particle composition (Sand, Silt, and Clay percentages), CaCO_3_ concentration (CaCO_3_), soil organic C concentration (SOC), soil total N concentration (TN), soil total P concentration (TP). *, p < 0.05; **, p < 0.01.*

*Figure S8. Distribution of the proportion of reference genomes per genus in the GTDB r214 database.*

*Figure S9. Global distribution of the uncertainty in prediction of uncultivated taxa. Uncertainty is represented by standard deviation (SD) of the predictions using random-forest models based on 24 environmental factors. All projections are displayed on a 0.1* × *0.1° scale of latitude and longitude. Areas of water, permanent wetlands, croplands, urban/built-up areas, and snow/ice areas were excluded from the projections.*
