# Supplementary material for: Genomic characteristics and geographical distribution of uncultivated soil prokaryotes
Source: BMC Genomics. 2026 Aug 4;27:655. doi: 10.1186/s12864-026-13092-0 (PMC13445660; doi:10.1186/s12864-026-13092-0)
Supplement: Supplementary file 2 [file 12864_2026_13092_MOESM2_ESM.docx]

Table S1. Description of data for the 24 predictors of soil uncultivated proportion on a global scale.

| **Factor category** | **ID** | **Variable** | **Unit** | **Source** | **Period/layer** | **Reference** |
| --- | --- | --- | --- | --- | --- | --- |
| **Climate** | 1 | Annual mean temperature (MAT) | °C | WorldClim2 | 1970-2000 | Fick and Hijmans, 2017 |
|  | 2 | Annual mean precipitation (MAP) | mm | WorldClim2 | 1970-2000 | Fick and Hijmans, 2017 |
|  | 3 | Temperature seasonality (TSEA) | 10^-2^ °C | WorldClim2 | 1970-2000 | Fick and Hijmans, 2017 |
|  | 4 | Precipitation seasonality (PSEA) | % | WorldClim2 | 1970-2000 | Fick and Hijmans, 2017 |
|  | 5 | Aridity index (AI) | - | global-aridity-index-and-potential-evapotranspiration-climate-database-v2/ | 1970-2000 | Trabucco and Zomer, 2018 |
| **Vegetation** | 6 | Shannon diversity enhanced vegetation index (EVI) | - | http://www.earthenv.org/texture | - | Tuanmu and Jetz, 2015 |
|  | 7 | Leaf area index (LAI) | m^2^ m^-2^ | https://daac.ornl.gov/cgi-bin/dsviewer.pl?ds_id=1653 | 1981-2015 | Mao and Yan, 2019 |
|  | 8 | Gross primary production (GPP) | g C m^-2^ d^-1^ | https://daac.ornl.gov/cgi-bin/dsviewer.pl?ds_id=1789 | 1982-2016 | Madani and Parazoo, 2020 |
|  | 9 | Aboveground biomass (AGB) | Mg C ha^-1^ | https://daac.ornl.gov/VEGETATION/guides/Global_Maps_C_Density_2010.html | 2010 | Spawn et al., 2020 |
|  | 10 | Belowground biomass (BGB) | Mg C ha^-1^ | https://daac.ornl.gov/VEGETATION/guides/Global_Maps_C_Density_2010.html | 2010 | Spawn et al., 2020 |
|  | 11 | Litter production (Litter) | g C m^-2^ y^-1^ | Provided by the authors | 1982-2016 | He et al., 2021 |
| **Topography** | 12 | Elevation | m | http://www.earthenv.org/topography | - | Amatulli et al., 2018 |
|  | 13 | Slope | degrees | http://www.earthenv.org/topography | - | Amatulli et al., 2018 |
| **Soil** | 14 | Depth to Bedrock | cm (≤ 200) | https://files.isric.org/soilgrids/former/2017-03-10/data/ | - | Hengl et al., 2017 |
|  | 15 | Soil clay percentage (Clay) | % of weight | http://globalchange.bnu.edu.cn/research/soilw#download | 0-10 cm |  |
|  | 16 | Soil silt percentage (Silt) | % of weight | http://globalchange.bnu.edu.cn/research/soilw#download | 0-10 cm |  |
|  | 17 | Soil sand percentage (Sand) | % of weight | http://globalchange.bnu.edu.cn/research/soilw#download | 0-10 cm |  |
|  | 18 | Soil moisture (Moisture) | % of volume | http://globalchange.bnu.edu.cn/research/soilw#download | 0-10 cm |  |
|  | 19 | Cation exchange capacity (CEC) | cmol kg^-1^ | http://globalchange.bnu.edu.cn/research/soilw#download | 0-10 cm |  |
|  | 20 | Soil pH in H_2_O (pH) | - (*10) | http://globalchange.bnu.edu.cn/research/soilw#download | 0-10 cm |  |
|  | 21 | CaCO_3_ | % of weight (*10^2^) | http://globalchange.bnu.edu.cn/research/soilw#download | 0-10 cm |  |
|  | 22 | Soil organic C concentration (SOC) | % of weight (*10^2^) | http://globalchange.bnu.edu.cn/research/soilw#download | 0-10 cm |  |
|  | 23 | Soil total N concentration (TN) | % of weight (*10^2^) | http://globalchange.bnu.edu.cn/research/soilw#download | 0-10 cm |  |
|  | 24 | Soil total P concentration (TP) | % of weight (*10^2^) | http://globalchange.bnu.edu.cn/research/soilw#download | 0-10 cm |  |

**Reference for this Table**

G. Amatulli, S. Domisch, M. N. Tuanmu, B. Parmentier, A. Ranipeta, J. Malczyk, W. Jetz, A suite of global, cross-scale topographic variables for environmental and biodiversity modeling. *Sci. Data* **5,** 180040 (2018). https://doi.org/10.1038/sdata.2018.40.

S. E. Fick, R. J. Hijmans, WorldClim 2: new 1km spatial resolution climate surfaces for global land areas. *Int. J. Climatol.* **37,** 4302-4315 (2017).

Y. He, X. Wang, K. Wang, S. Tang, H. Xu, A. Chen, P. Ciais, X. Li, J. Peñuelas, S. Piao, Data‐driven estimates of global litter production imply slower vegetation carbon turnover. *Global Change Biol.* **27,** 1678-1688 (2021).

T. Hengl, J. M. de Jesus, G. B. M. Heuvelink, M. R. Gonzalez, M. Kilibarda, A. Blagotić, et al., SoilGrids250m: Global gridded soil information based on machine learning. *PLoS ONE* **12,** e0169748 (2017).

N. Madani, N. C. Parazoo, Global Monthly GPP from an Improved Light Use Efficiency Model, 1982-2016. ORNL DAAC, Oak Ridge, Tennessee, USA. (2020). https://doi.org/10.3334/ORNLDAAC/1789.

J. Mao, B. Yan, Global Monthly Mean Leaf Area Index Climatology, 1981-2015. ORNL DAAC, Oak Ridge, Tennessee, USA (2019). https://doi.org/10.3334/ORNLDAAC/1653.

S. A. Spawn, H. K. Gibbs, Global Aboveground and Belowground Biomass Carbon Density Maps for the Year 2010. ORNL DAAC, Oak Ridge, Tennessee, USA (2020). https://doi.org/10.3334/ORNLDAAC/1763.

A. Trabucco, R. J. Zomer, Global Aridity Index and Potential Evapo-Transpiration (ET0) Climate Database v2. CGIAR Consortium for Spatial Information (CGIAR-CSI) (2018).

M. N. Tuanmu, W. Jetz, A global, remote sensing‐based characterization of terrestrial habitat heterogeneity for biodiversity and ecosystem modelling. *Global Ecol. Biogeogr.* **24,** 1329-1339 (2015). https://doi.org/10.1111/geb.12365.
